# Supplementary material for: Whole transcriptome expression profiles in kidney samples from rats with hyperuricaemic nephropathy
Source: PLoS One. 2022 Dec 19;17(12):e0276591. doi: 10.1371/journal.pone.0276591 (PMC9762607; doi:10.1371/journal.pone.0276591)
Supplement: S1 Table — (DOCX) [file pone.0276591.s001.docx]

**Whole transcriptome expression profiles in** **kidney samples from rats with** **hyperuricaemic** **nephropathy**

Na Li*, Mukaram Amatjan*, Pengke He*, Meiwei Wu, Hengxiu Yan, Xiaoni Shao#

College of Pharmacy, Southwest Minzu University, Chengdu 610041, China

* These authors contributed equally: Na Li, Mukaram Amatjan and Pengke He

#Correspondence should be addressed to Xiaoni Shao

**S1 Table. Comprehensive list of all primer sequences used.**

| **Name** | **Sequence (Forward)** | **Sequence (Reverse)** |
| --- | --- | --- |
| β-Actin | TGTCACCAACTGGGACGATA | GGGGTGTTGAAGGTCTCAAA |
| Ptafr | TATGTGCTGTGGGTCTTTGC | AGTCGCCCTCGTTGGAGTA |
| Adam19 | AGAGACCTGCTACACCGCAAG | ACTTTCCTCACAGTCCCGTGGT |
| A2m | CTCTCAGCAGCAGAAGGACAATGG | CTATGGTGATGTAGGCAGACAAGGTG |
| Nlrp12 | CCCCAGATAACCTACAAAGAC | AGCCGAGTGTAACGATGAC |
| Actin | TGTCACCAACTGGGACGATA | GGGGTGTTGAAGGTCTCAAA |
| LOC102547703 | CAGGGACTTAGCCAATGAACGAGAC | AGACAGACAGAGACAGACAGACAGAC |
| LOC102555374 | ACTTGTCAAATGTCTGCCTCTTCCTC | TCAACTCCACCTGAACAGTCACAATC |
| U6 | CTCGCTTCGGCAGCACA | AACGCTTCACGAATTTGCGT |
| U6 RT | TGGTGTCGTGGAGTCG | |
| Mir-351-5p | TCCCTGAGGAGCCCTTTGA | AGTGCAGGGTCCGAGGTATT |
| Mir-351-5p RT | GTCGTATCCAGTGCAGGGTCCGAGGTATTCGCACTGGATACGACTCAGGC | |
| Mir-760-5p | GCCCCTCAGGCCACCAG | AGTGCAGGGTCCGAGGTATT |
| Mir-760-5p RT | GTCGTATCCAGTGCAGGGTCCGAGGTATTCGCACTGGATACGACCGGGCT | |
