# Supplementary material for: Whole transcriptome expression profiles in kidney samples from rats with hyperuricaemic nephropathy
Source: PLoS One. 2022 Dec 19;17(12):e0276591. doi: 10.1371/journal.pone.0276591 (PMC9762607; doi:10.1371/journal.pone.0276591)
Supplement: Attachment — Submitted filename: Response to Reviewers.docx [file pone.0276591.s011.docx]

**Response to Reviewers**

Dear Editor and Reviewers,

Thank you for taking time out of your busy schedule to review the manuscript again. These comments are all valuable and helpful for improving our manuscript, as well as the important guiding significance to our research. We have studied comments carefully and have made correction which we hope meet with approval. And we use the “Track Changes” option in Microsoft Word to show the revised portions, deleted content is shown with a red strikeout and added content is shown with a red underscore. The revision instructions are as follows:

Summary of the revision：

- Discussion: We removed the unreasonable parts to make the sentence more coherent, and also deleted their references accordingly.
- Figure: We have made changes to all figures to make text labels and legends clearer. The range of the Y axis has also been adjusted to highlight the differences.

**Responses to the reviewers’ and editorial comments**

**(Response: answers from the authors are in** blue color**, the figures that are just used in the response are named Figure R+number or Table R+number)**

Priyadarshini Kachroo

Academic Editor

**Comment 1.** We would need high quality main figures to represent the data for acceptance of the manuscript. Figure 1A-C can be considered as the labels are bigger.

**Response:** Thanks for your comments. We have carefully revised the figure.

**Comment 2**. However, currently, it is difficult to read the text of the Figure 2 as well as the labels/legends clearly.

**Response:** Thank you for your significant reminder. The pictures have been corrected.

**Comment 3.** This is true for Figure 3A-B and Figure 4 all panels.

**Response:** Thank you for pointing this out. We checked the figures and modified the label/legend of the figures.

**Comment 4.** Figure 5 is hairball but it would be nice if we can get a better resolution image.

**Response:** We have uploaded the figures and adjusted the resolution of figure to 600 dpi according to the requirement of the ***Plos one***.

**Comment 5.** Figure 6 needs clear labels of the legend and also titles on x and y-axis.

**Response:** Thanks for the reminder. We modified the legend labels in Figure 6 and the titles on the x- and y-axis to make them clearer.

**Comment 6.** Figure 7 legends are totally not readable as well as the text inside the nodes/ circles of the figure.

**Response:** We apologize that our figures are unreadable. And we have modified Figure 7 based on the editor's suggestion.

**Comment 7.** Similar to Figure 4, Figure 8 axis-labels, title and legend need to be made clear. They are difficult to understand even after zooming in.

**Response:** Thank you for your valuable comments. We modified the axis labels, title and legend in Figure 8.

**Comment 8.** Figure 9 can be accepted as is, but if possible, please make the text labels clear.

**Response:** Thank you for your advice. We have modified the text labels in Figure 9.

**Reviewer #2:** 
**Comment 1.** Regarding comment 1, how about displaying the data as a bar plot with dots? And it would be better to adjust the range of y-axis for highlighting the differences.

For example, y axis could be narrowed down from 0.8 to 1.5 for Nlrp12.

**Response:** Thank you for pointing this out. According to your requirements, we have reduced the y-axis, shrunk the y-axis of Figures 9B, C, and H from 1.5 to 1.2, and the y-axis of Figure 9G from 2 to 1.8, making the difference look more obvious in the figure.

**Comment 2.** Regarding comment 3, the authors modified the explanation in page 27, line 563-565 as follows.

"PAF is a 562 phospholipid that plays an important role in tumour transformation, tumour growth, angiogenesis, 563 metastasis and pro-inflammatory processes[63]. Haemorrhagic fever[64] and bacterial sepsis[65]"

As far as I understand, "Haemorrhagic fever[64] and bacterial sepsis[65]" is not a sentence, so it is hard to get what it means.

**Response:** We are sorry for our carelessness and we have corrected the mistakes in the revised manuscript (page **27**, line **563**) and adjusted the order of the references. Thanks for your reminder.
